# Blood and guts: The intestinal vasculature during health and helminth infection

**DOI:** 10.1371/journal.ppat.1007045

**Published:** 2018-07-19

**Authors:** Maria E. Gentile, Irah L. King

**Affiliations:** 1 Meakins-Christie Laboratories, Department of Microbiology and Immunology, McGill University Health Centre Research Institute, Montreal, Québec, Canada; 2 Meakins-Christie Laboratories, Department of Medicine, McGill University Health Centre Research Institute, Montreal, Québec, Canada; University of Wisconsin Medical School, UNITED STATES

## What is the role of the intestinal vasculature during homeostasis?

The gastrointestinal tract is home to a complex ecosystem of host–microbial interactions that not only serve to extract dietary nutrients and remove waste but also to promote the development of the immune system. These metabolic and host defense signals must be distributed systemically to ensure proper organ function and immunosurveillance, a process largely achieved by the intestinal vascular system.

Since the vasculature is tasked with circulating blood from a central pump (the heart) to every tissue, it must be intricately branched. Leaving the left ventricle of the heart, the abdominal aorta divides into multiple branches, three of which supply the gastrointestinal system with oxygenated blood. The celiac trunk supplies blood to the stomach, and the superior and inferior mesenteric arteries supply the small intestine, the proximal colon and distal colon, respectively. These arteries further divide into arterioles extending to the tip of each individual villus. In the villus centre, the arteriole branches into capillaries that allow oxygenated blood to flow into the gut while simultaneously absorbing CO_2_ and nutrients [1]. Following this exchange, blood flows into venules that lead to the hepatic portal venous system, which drains into the inferior vena cava before returning to the heart [1].

Regulation of blood flow in the intestine is a highly dynamic and precise process. During feeding, partially digested food (chyme) is generated in the stomach and flows to the small intestine. As chyme passes through the small intestine, blood flow increases to that segment of the gut [2]. Once nutrient absorption has occurred, the blood flow in that area returns to baseline [2]. Interestingly, this change in blood flow is influenced by the nutrient composition of the chyme and not simply gut distension [3]. Simultaneously, digested food products are detected by chemosensory enteroendocrine cells and absorptive enterocytes [4], specialized subsets of intestinal epithelial cells (IECs) that transport nutrients across the epithelial monolayer into the lamina propria where they are ferried to the fenestrated blood endothelium for systemic distribution [5]. These nutritive functions are complemented by the transport of microbial-derived products that locally condition circulating leukocytes or act in distal tissues such as the bone marrow, the site of adult hematopoiesis [6].

## How does the vasculature change upon intestinal infection or injury?

**Fig 1:**
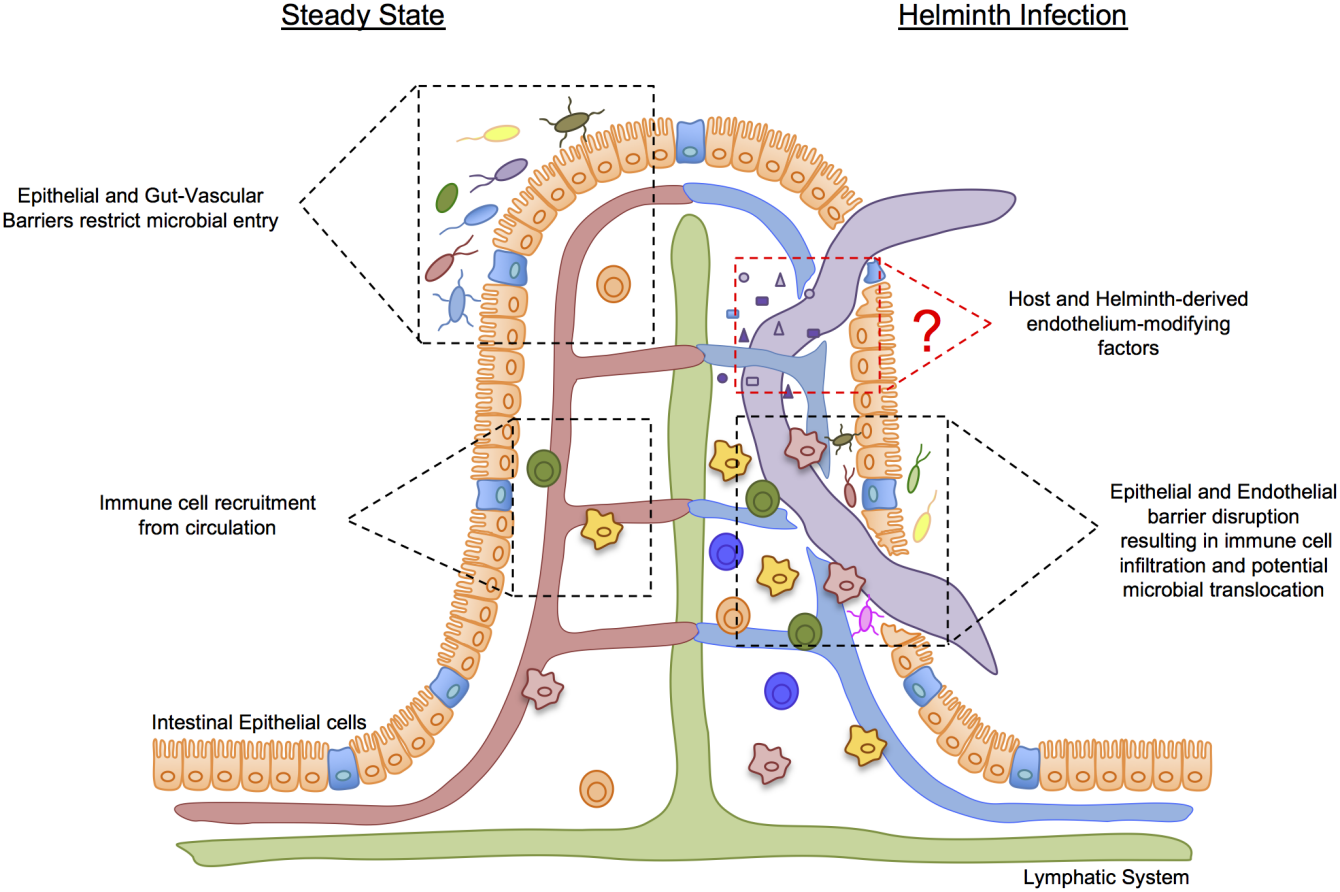
The multi-tasking nature of the intestinal vasculature. At steady state, the intestinal epithelial barrier and the GVB control systemic microbial dissemination and the recruitment of circulatory cells to maintain a tolerogenic environment. During the tissue-invasive stages of diverse parasitic helminth species, a presumed loss of epithelial and endothelial barrier integrity results in a rapid and robust accumulation of inflammatory cells and tissue damage [8]. In this context, both helminth- and host-derived factors contribute to establishing a state of vascular tolerance to limit tissue damage and promote repair. GVB, gut-vascular barrier.

The gastrointestinal tract is well characterized for its physical barrier composed of a single epithelial cell layer separating the luminal microbes in contact with their apical surface from the immune cells found within the gut parenchyma (Fig 1). As such, a high degree of mucosal immune regulation is needed to maintain homeostasis of the gut. To this end, IECs detect pathogenic motifs via pattern recognition receptors and, in turn, produce anti-microbial factors and mucous that limit microbial invasion. IECs also promote immune regulation by facilitating the development of T regulatory cells and tolerogenic dendritic cells and macrophages via production of thymic stromal lymphopoietin, retinoic acid, and transforming growth factor-β (TGF) [7].

Just as IECs must carefully regulate what enters the host tissue, the intestinal vasculature must also be highly selective in what enters the circulation. The Rescigno group recently provided evidence of a gut-vascular barrier (GVB) with phenotypic and functional similarities to the blood–brain barrier of the central nervous system [9] (Fig 1). Upon injection of fluorescently labeled dextran into the intestinal lumen, they observed leakage into the circulation during infection with *Salmonella enterica* serovar Typhimurium that was not detectable under steady state conditions. This change of barrier permeability was due to *S*. *enterica*-induced down-regulation of endothelial Wnt signalling [9]. Decreased Wnt signalling was associated with an increase in plasmalemma vesicle-associated protein (PLVAP), a molecular component of fenestrated endothelium required for vascular integrity [5]. This study sets the stage for further investigations into the relatively unknown molecular and microbial regulation of the GVB and its impact on intestinal and systemic health.

In response to pathogen invasion or loss of barrier integrity, both IECs and tissue-resident leukocytes secrete a host of factors such as cytokines, reactive oxygen species, and lipid mediators that increase endothelial cell expression of chemokine receptors and integrins that promote immune cell extravasation into the lamina propria. Inflammatory mediators also increase the vascular permeability that further facilitates both inter- and trans-cellular diapedesis [10]. Within the tissue, immune cells encounter effector-enhancing cytokines and pathogen-derived products that amplify the inflammatory response and neutralize (via antibody production) or kill (via phagocytosis or cell lysis) invading pathogens. In the case of infection by nonreplicating, multicellular organisms such as parasitic helminths, the focus of the immune response must be to limit tissue damage and tolerate host invasion (Fig 1). Although endothelial cells reciprocally produce oxidants that can either enhance or counteract cell stress associated with inflammation, how the intestinal vasculature responds to distinct forms of infection and injury and shapes the resulting immune response is largely unknown.

## Does helminth infection influence the host intestinal vasculature?

The term “helminth” is a nontaxonomical word used to classify macroscopic parasitic worms that have a life stage outside of their primary host. Intestinal helminth infections are neglected tropical diseases that infect more than 1 billion people that primarily reside in the developing world [11]. The majority of helminths are soil-transmitted parasites including hookworms (e.g., *Necator americanus*), roundworms (e.g., *Ascaris lumbricoides*), and whipworms (e.g., *Trichuris trichiuria*) [11].

The relationship between the intestinal vasculature and helminth infection was initially observed in 1880 by Edoardo Perroncito, who noted severe malnutrition and anemia among workers during a severe outbreak of parasite infection [12]. Although one of the most frequent morbidities of helminth infection is intestinal bleeding [13], it is important to note that each type of helminth has unique routes of migration through its host. Thus, the tissue damage and requirements for host defense are unique to each parasite. Nevertheless, most intestinal-dwelling helminths must break the epithelial barrier and enter host tissue for either maturation and/or feeding purposes. As such, capillaries and arterioles within the intestinal tissue are mechanically ruptured, leading to blood loss (Fig 1). Indeed, the severity of blood loss positively correlates with parasite burden [14]. In addition, intestinal helminths release proteases, hyaluronidase, and anticlotting factors that break down the extracellular matrix of the gut and further compromise vascular integrity [15, 16].

Blood loss due to helminth infection frequently results in iron-deficient anemia. During infection with *A*. *duodenale* and *N*. *americanus*, the blood loss per day is approximately 0.2 mL and 0.15 mL, respectively [17]. In a systematic review of 14 randomized controlled trials comparing the effects of antihelminthic drugs on haemoglobin levels, Gulani and colleagues found moderate increases in the group receiving treatment, suggesting a basis for the decreased prevalence of anemia in South Asia and Africa [18]. These data support the concept that helminth infections have a negative impact on the intestinal vasculature.

## Does the immune system impact vascular tolerance to helminth infection?

Helminth-induced vascular damage can have severe consequences on nutritional status and growth but rarely results in irreversible damage and mortality [11]. This observation suggests that, despite the chronic nature of helminth infection, the host has evolved mechanisms to limit the vascular damage incurred. The phenomenon whereby the host protects itself from excessive tissue damage or immunopathology without directly affecting pathogen burden is known as disease tolerance. The precise mechanisms that allow the host to tolerate vascular damage during helminth infection are poorly understood. However, hints from other studies suggest that the immune system makes an important contribution to vascular physiology and tolerance. For example, innate immune cells such as macrophages and natural killer cells have been shown to play a key role in the maintenance of vascular integrity during intracerebral hemorrhage and pregnancy, respectively [19, 20]. Whether these or other leukocyte subsets regulate the integrity of the GVB during intestinal injury or infection remains an open area of investigation.

## Concluding remarks

The intestinal vasculature is crucial for nutrient absorption, as a barrier against microbes, and for the recruitment and conditioning of immune cells. The recent discovery of the GVB presents an exciting opportunity to understand how the unique structure of the gut endothelium responds to diverse insults and the cellular networks that promote vascular tolerance and repair. It is intriguing to consider how both host and pathogen modulate the intestinal vasculature to their own benefit while continuing to tolerate one another. However, the cellular and molecular changes that characterize vascular damage and repair during microbial and macrobial challenge remain largely unknown. Further studies into the mechanisms of intestinal vasculature control could open new avenues for therapeutic interventions that limit the morbidity associated with intestinal helminth infection and other settings of vascular damage, such as inflammatory bowel disease.
